# Supplementary material for: Responses of Arbuscular Mycorrhizal Fungi and Plant Communities to Long-Term Mining and Passive Restoration
Source: Plants (Basel). 2025 Feb 14;14(4):580. doi: 10.3390/plants14040580 (PMC11859955; doi:10.3390/plants14040580)
Supplement: Supplementary file 1 [file plants-14-00580-s001.zip › plants-3463098-supplementary.pdf]

Supplementary Materials

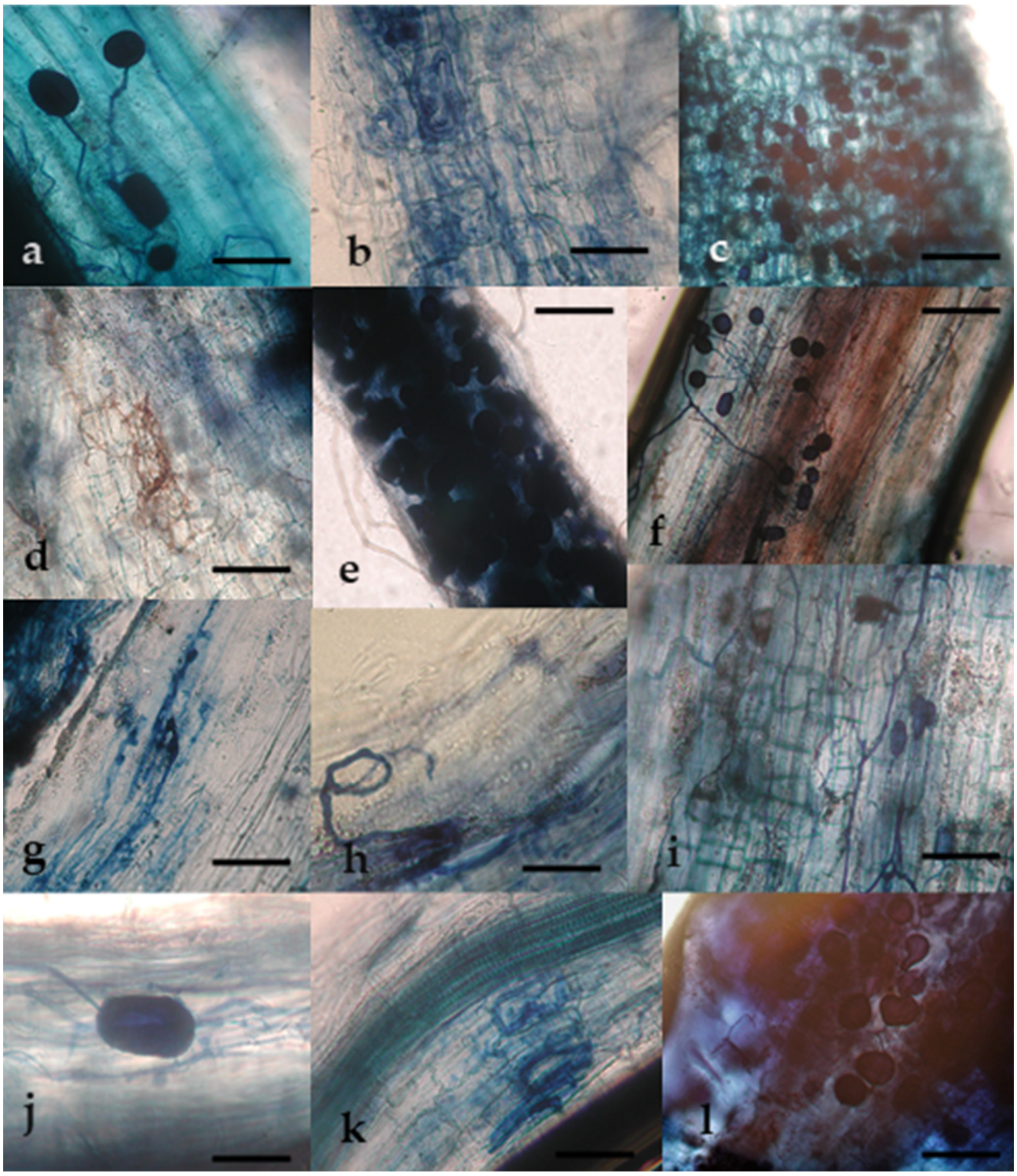

Supplemental Figure S1: Arbuscular mycorrhizal (AM) colonisation in roots of plants from Paramillos de Uspallata mine and an area outside mine. Detail of stained roots from Urban ruins area (a-f), Exploitation area 1 (g,h), Exploitation area 2 (i,j) and Off-mine area (k,l): *Pappostipa speciosa* colonised by AM fungi, bar= 150 μm (a); Paris type colonisation in *P. speciosa,* bar= 20 μm (b); intraradical spores in roots of *Fabiana patagonica,* bar= 300 μm (c); melanized hyphae of other fungal endophytes in roots of *Phacelia sinuata,* bar= 200 μm (d); intraradical spores in *Junellia uniflora,* bar= 100 μm (e); Arum type colonisation and other fungal endophyte in *Maihueniopsis glomerata,* bar= 300 μm (f); hyphopodium and hyphal development of AM fungi in roots of *Adesmia horrida,* bar= 100 μm (g)*;* Hyphopodium of AM fungi in roots of *Senecio uspallatensis,* bar= 20 μm (h); hyphal development of AM fungi in roots of *Tetraglochin alata*, bar= 300 μm (i); Arum type colonisation in roots of *Pappostipa* sp., bar= 50 μm (j); Paris type AM colonisation in roots of *Artemisia mendozana,* bar= 20 μm (k); intraradical spores in *Viola atropurpurea*, bar= 100 μm (l).

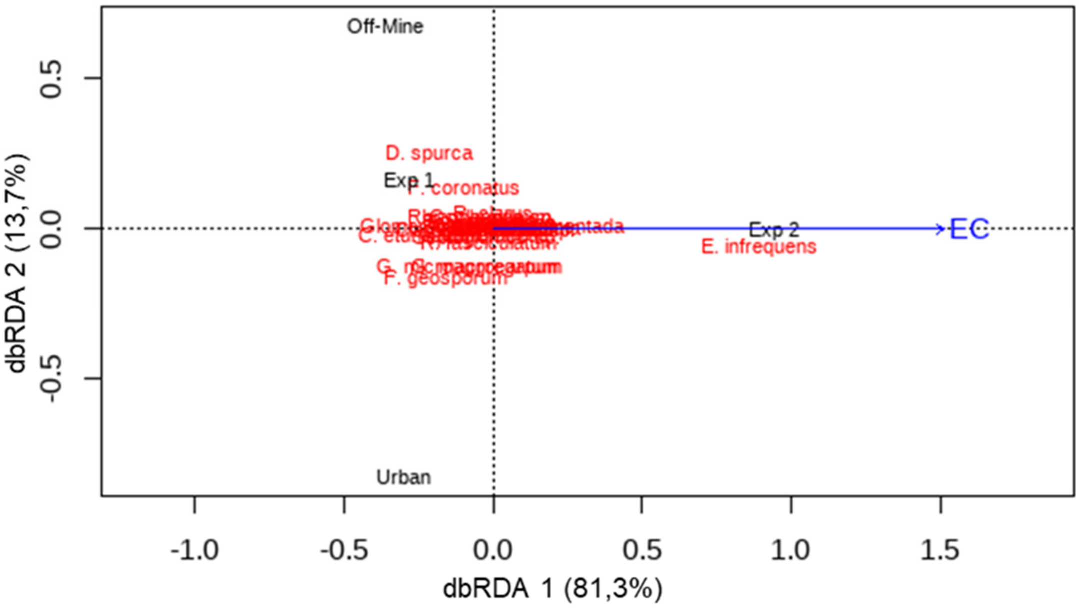

Supplemental Figure S2: Distance-based Redundancy Analysis (dbRDA) considering the different areas within Paramillos de Uspallata mine and an outside mine, the relative abundance of each AM fungal species, and the physicochemical variables of the soils. Urban: urban ruins area; Exp 1: Exploitation area 1; Exp 2: Exploitation area 2; Off-mine: off-mine area. EC: Electrical conductivity.
